# Antiplatelet Activity of Tetramethylpyrazine via Regulation of the P2Y12 Receptor Downstream Signaling Pathway

**DOI:** 10.1155/2022/7941039

**Published:** 2022-03-26

**Authors:** Baoyi Guan, Jie Gao, Yu Tan, Xiaojuan Ma, Dazhuo Shi

**Affiliations:** ^1^China Heart Institute of Chinese Medicine, China Academy of Chinese Medical Sciences, Beijing 100091, China; ^2^Cardiovascular Diseases Center, Xiyuan Hospital, China Academy of Chinese Medical Sciences, Beijing 100091, China; ^3^National Clinical Research Center for Chinese Medicine Cardiology, Xiyuan Hospital, China Academy of Chinese Medical Sciences, Beijing 100091, China

## Abstract

**Background:**

Tetramethylpyrazine (TMP) is an alkaloid in Chinese herbal medicine, which possesses antiplatelet activity. TMP inhibits platelet activation in many ways. The platelet P2Y_12_ receptor for adenosine 5′ diphosphate (ADP) plays a central role in platelet function, hemostasis, and thrombosis. Here, we investigated the inhibitory effect of TMP on P2Y_12_ receptor-related platelet function.

**Methods:**

The inhibitory potential of TMP was assessed using agonist-induced platelet aggregation, flow cytometric analysis of CD62p expression, PAC-1 activation, and fibrin clot retraction. After the P2Y12 receptor-related signaling pathway was inhibited using the blocker, platelet activation was studied by platelet aggregation, CD62p expression, and PAC-1 activation. The secretion of cyclic adenosine monophosphate (cAMP) was measured using enzyme-linked immunosorbent assay (ELISA), and the expression of signaling pathway protein, phosphorylation of vasodilator-stimulated phosphoprotein, and phosphorylation of Akt were investigated using western blotting. The release of platelet inflammatory mediators was measured using ELISA.

**Results:**

TMP had an antiplatelet effect by inhibiting ADP-induced aggregation, P-selectin secretion, and glycoprotein (GP) IIb/IIIa expression and reducing the release of atherosclerotic-related inflammatory mediators (sCD40L and IL-1*β*). TMP decreased the area of clot retraction, reflecting inhibition of GPIIb/IIIa activation. TMP inhibited adenosine diphosphate-induced platelet activation via increased cAMP production, VASP^ser157^ phosphorylation, and Akt dephosphorylation.

**Conclusion:**

TMP selectively inhibits ADP-induced platelet activation via P2Y_12_ receptor-related signaling pathways.

## 1. Introduction

The prevalence and mortality of myocardial infarction, ischemic stroke, and other cardiovascular and cerebrovascular diseases increase worldwide [1]. The so-called atherothrombotic process is the common pathogenesis, characterized by rupture or erosion of vulnerable plaques and the subsequent activation of the clotting cascade; platelets are the critical mediators [2]. Antiplatelet agents, especially the P2Y_12_ antagonist clopidogrel, help patients with evidence of atherothrombotic diseases [3, 4]; nevertheless, outcomes remain poor. The causes underlying this phenomenon may be adverse effects such as gastrointestinal disorders and internal bleeding [5]. Several studies showed that about 30% of patients with atherothrombotic diseases were low responders/nonresponders to clopidogrel, leading to higher ischemic risk [6–8]. For these reasons, it is essential to study antiplatelet treatments and develop novel P2Y12 antagonists with increased efficacy and safety profiles.

Platelets are activated by extracellular stimuli (e g., thrombin, collagen, and ADP) and their specific receptor signaling pathways [9]. Central among these stimuli is ADP, a weaker platelet agonist than thrombin and collagen, which induces several platelet responses and potentiates platelet aggregation to other agonists [10]. ADP stimulates platelet activation through interaction with the Gaq-coupled P2Y_1_ and Gai-coupled P2Y_12_ receptors. ADP-induced P2Y_1_ signaling is responsible for the elevation of cytosolic Ca^2+^ and altering the platelet shape [11]. P2Y_12_ signaling modulates aggregation through inhibition of adenylyl cyclase (AC), resulting in reduced cAMP concentrations, which mediates dephosphorylation of vasodilator-stimulated phosphoprotein (VASP) [12]. Hence, dephosphorylation of VASP induced by ADP through P2Y_12_ receptor activation might enhance platelet activation. In this regard, the phosphorylation or dephosphorylation levels of VASP might reflect P2Y_12_ inhibition or activation state, which might serve as a marker to measure the efficacy of clopidogrel treatment. In addition to the inhibition of AC, P2Y_12_ potentiates platelet release and aggregation via activating phosphoinositide-3 kinase (PI3K)/Akt signaling [12]. Thus, the P2Y_12_ receptor and downstream signaling appear to be responsible for the efficacy of antiplatelet agents.

There is growing interest in natural bioactive components that suppress platelet function isolated from Chinese herbs and natural plants [13]. Interestingly, some may exert an inhibitory effect on platelet activation by modulating membrane receptor-related signaling pathways. *Ligusticum chuanxiong* (LC) is a traditional Chinese medicine that activates blood circulation and removes blood stasis. Pharmacological studies showed that LC dilates coronary arteries and exerts antithrombotic, antioxidant, sedative, and bacteriostatic effects [14]. These effects are attributed to the phytochemical compounds of LC, of which alkaloids are the primary bioactive constituents. Studies demonstrated that tetramethylpyrazine (TMP) extracted from LC alleviates platelet-mediated thrombotic disease by suppressing the release of thromboxane A2 and promoting prostacyclin production [15]. TMP suppressed intracellular Ca^2+^ mobilization in a concentration-dependent manner and inhibited platelet aggregation [16]. Nevertheless, the mechanisms by which this inhibition occurs have not been thoroughly investigated. Regarding the effects of TMP on platelet function, we examined the effects of TMP on the P2Y_12_ receptor-dependent platelet aggregation and explored the detailed mechanisms of TMP-mediated inhibition of platelet activation via the P2Y_12_-related signaling pathway.

## 2. Materials and Methods

### 2.1. Agents

Tetramethylpyrazine (TMP) was obtained from Shanghai Yuanye Bio-Technology Co., Ltd. (Shanghai, China). Ticagrelor (Tica) was from Selleck (Shanghai, China). Adenosine 5'-diphosphate (ADP) and MRS2179 (P2Y_1_ receptor antagonist) were purchased from Sigma-Aldrich (St. Louis, MO, USA). Thrombin was from Beijing Solarbio Science & Technology Co., Ltd. (Bejing, China). Dimethyl sulfoxide (DMSO) was from Amresco (Houston, Texas, USA). Antibodies (anti-CD61-APC, anti-CD62P-phycoerythrin-labeled (PE), and anti-GPIIb/IIIa-FITC PAC-1) were purchased from BD Biosciences (San Diego, CA, USA). SQ22536 (an AC inhibitor) and LY294002 (a PI3k inhibitor) were obtained from APExBIO Technology LLC (Houston, Texas, USA). The cyclic AMP enzyme immunoassay (ELISA) kit was from Cayman Chemical (Ann Arbor, MI, USA). The IL-1*β* and soluble CD40 ligand (sCD40L) ELISA kits were obtained from Elabscience Biotechnology Co., Ltd. (Wuhan, China). The bicinchoninic acid (BCA) protein assay kit was from Beyotime (Shanghai, China). Antibodies against phospho-VASP^ser157^, Akt, phospho-Akt^ser473^, and phospho-Akt^Thr308^ were acquired from Cell Signaling Technology (Beverly, MA, USA). Tetramethylpyrazine, SQ22536, and LY294002 were dissolved in DMSO for further use in experiments. DMSO 0.1% was employed as the vehicle.

### 2.2. Platelets Preparation

The Ethics Committee of Xiyuan Hospital, China Academy of Chinese Medical Sciences approved the study protocol (2018XLA067-3). After receiving written informed consent, venous blood samples were taken from young, healthy volunteers who had not taken any anticoagulation or antiplatelet agents in the month before the study. The samples were collected by phlebotomy with a vacuum tube system and placed in 3.2% citrate tubes (9:1 v/v) (Becton Dickinson Vacutainer Systems, Franklin Lakes, NJ, USA). Platelet-rich plasma (PRP) was prepared by centrifuging the blood samples at 700 rpm for 10 min, and two-thirds of PRP was removed to a centrifuge tube. The remaining blood samples were centrifuged at 3000 rpm for 10 min to prepare platelet-poor plasma (PPP). The PRP was adjusted to a final concentration of 2.0–2.5 × 10^8^ platelets/mL by PPP.

The PRP was centrifuged at 3000 rpm for 10 min to obtain the platelet sediment. Platelet sediment was then washed with washing buffer. Washed platelets were then gently resuspended in Tyrode's-HEPES buffer containing ACD anticoagulant to a final concentration of 2.0–2.5 × 10^8^ platelets/mL.

### 2.3. Platelets and Grouping

To investigate the importance of P2Y12 receptors and the related signaling pathways in the antiplatelet activity of TMP, we used the P2Y1 antagonist MRS2179 (100 *μ*M) to inhibit the relative activity of the platelet ADP receptor P2Y1 [17]. Before the study, 12 separate platelet donors were randomly divided in a 1:1 ratio into two independent experiments.

In the first experiment, we tested the effect of TMP on the three primary functions of platelets: aggregation, secretion, and adhesion. Platelets were randomly allocated as follows: (1) resting group (platelets cultured at 37°C for 10 min), (2) control group (platelets exposed only to ADP), (3) model group (platelets were pretreated with MRS2179 for 5 min and then treated with the vehicle for 5 min before adding ADP), (4) Tica group (platelets were pretreated with MRS2179 for 5 min and then treated with 100 nM Tica for 5 min before adding ADP), and (5) TMP groups (platelets were pretreated with MRS2179 for 5 min and then treated with 1–3 mM TMP for 5 min before adding ADP). After various interventions, platelets were incubated at 37°C.

**Figure 1:**
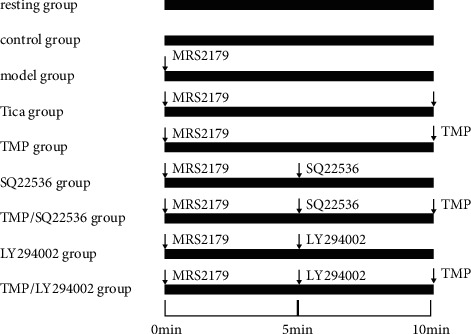
Experimental plan for the assessment of P2Y_12_ receptor-mediated platelet activation during TMP treatment.

In the second experiment, to further determine whether the effect of TMP on platelet activation was specific to the P2Y12 signalings, we targeted the AC and PI3k pathways using the AC inhibitor SQ22536 (100 *μ*M) and the PI3k inhibitor LY294002 (10 *μ*M) (Figure 1). Platelets were randomly divided into nine groups as follows: (1) resting group (platelets were cultured at room temperature for 15 min), (2) control group (platelets were exposed only to ADP), (3) model group (platelets were pretreated with MRS2179 for 5 min and then treated with the vehicle for 10 min before adding ADP), (4) Tica group (platelets were pretreated with MRS2179 for 5 min and then treated with the vehicle for 5 min and 100 nM Tica for 5 min before adding ADP), (5) TMP group (platelets were pretreated with MRS2179 for 5 min and then treated with the vehicle for 5 min and 3 mM TMP for 5 min before adding ADP), (6) SQ22536 group (platelets were pretreated with MRS2179 for 5 min and then treated with the SQ22536 for 5 min and the vehicle for 5 min before adding ADP), (7) TMP/SQ22536 group (platelets were pretreated with MRS2179 for 5 min and then treated with the SQ22536 for 5 min and 3 mM TMP for 5 min before adding ADP), (8) LY294002 group (platelets were pretreated with MRS2179 for 5 min and then treated with the LY294002 for 5 min and the vehicle for 5 min before adding ADP), and (9) TMP/LY294002 group (platelets were pretreated with MRS2179 for 5 min and then treated with the LY294002 for 5 min and 3 mM TMP for 5 min before adding ADP). After the various interventions, platelets were incubated at 37°C.

### 2.4. Measurement of Platelet Aggregation

Platelet aggregation assays were performed using light-transmission aggregometry (Beijing Prisheng Instrument Co. Ltd., China) [18]. Before testing, the test hole of the platelet aggregometer was preheated to 37°C. After treatment with various interventions described in Section 2.3, 300 *μ*l of PRP were activated with 3 *μ*l ADP (20 *μ*M) and monitored for 5 min to obtain maximal aggregation values. Maximal aggregation% was defined as the increase of light transmission through PRP after adding agonist ADP compared to the baseline optical density set with PPP and was automatically calculated using a platelet aggregometer.

### 2.5. Measurement of CD62p Expression and PAC-1 Activation

CD62p (P-selection) surface expression and PAC-1 GPIIb/IIIa expression were measured using flow cytometry with P-phycoerythrin-labeled (PE) anti-CD62P and fluorescein isothiocyanate-labeled (FITC) anti-GPIIb/IIIa antibodies. Briefly, PRP was preincubated with various interventions. Then, 5 *μ*l PRP was added to flow tubes containing 100 *μ*l phosphate-buffered saline (PBS). After 5 min of stimulation at 37°C with ADP (20 *μ*M), platelets were incubated with anti-CD61-APC, anti-CD62-PE, and anti-GPIIb/IIIa-FITC PAC-1 for 20 min in the dark. After quench dilution with 0.5% paraformaldehyde for 10 min at 37°C, the samples were washed with PBS and resuspended in 500 *μ*l PBS. The samples were analyzed in a fluorescence-activated cell sorting flow cytometer (Beckman Coulter, Inc., CA, USA) according to the manufacturer's instructions. Platelets were gated using forward scatter and side scatter to distinguish CD61-positive platelets. Approximately, 10000 platelets were evaluated within this gate.

### 2.6. Assay of Platelet-Mediated Fibrin Clot Retraction

Human PRP was treated with various interventions at 37°C. Subsequently, clot retraction in human PRP was performed as described with minor modifications [19]. PRP (300 *μ*l) was mixed with 20 mM CaCl_2_ (20 *μ*l) and red blood cells (10 *μ*l) in an aggregometer tube. Then, Tyrode's-HEPES buffer was added to give the final volume of 1 mL. Thrombin (1 U/mL) was added to initiate fibrin clot formation, and then clot retraction was observed for 2 h at room temperature. Photographs of fibrin clots were recorded using a digital camera, and the area and retraction degree were measured using ImageJ software. Clot retraction was quantified by measuring the volume of extruded serum, and data were expressed as percentages.

### 2.7. Measurement of cAMP Levels in Platelets

cAMP measurement was performed using a cyclic AMP Select ELISA kit, following the manufacturer's instructions. The effect of TMP on cAMP platelet levels was evaluated in washed platelets after the incubation period and a 5 min stimulation with ADP. Then cold-ice HCl (0.1 M, 100 *μ*l) was added to washed platelets (100 *μ*l) to terminate the reaction. After centrifugation at 4°C at 10000 rpm for 10 min, precipitated proteins were removed and the supernatants were collected. The supernatants were stored at –80°C until performing the cAMP enzyme immunoassay.

### 2.8. Platelet Inflammatory Mediators

Platelet-related inflammatory mediators were measured in washed platelets after different interventions as described in Section 2.3. Washed platelets suspensions were stimulated with ADP for 5 min. Precipitated proteins were then removed by centrifugation at 10000 rpm at 4°C for 10 min, and the supernatants were collected and stored at −80°C until use. The concentrations of released IL-1*β* and sCD40L were determined using ELISA kits (Elabscience Biotechnology) according to the manufacturer's instructions.

### 2.9. Western Blot for the Expression of Akt, VASP-, and Akt-Phosphorylations

After platelet aggregation, the mixtures were centrifuged at 3000 rpm at 4°C for 15 min. Then supernatants were removed, and the pellets were washed twice with PBS and lysed on ice by adding an equal volume (100 *μ*l) of RIPA buffer (10 ml RIPA lysis buffer, 1 *μ*g/ml aprotinin, 1 mM protein phosphotyrosine phosphatase inhibitor, pH 7.5). Western bolt analysis was performed with the pellet. Briefly, the total protein concentration from the platelet was determined using a BCA protein assay kit. Proteins were resolved in 10% sodium dodecyl sulfate-polyacrylamide gel electrophoresis gels and were transferred onto polyvinylidene difluoride (PVDF) membranes. The membranes were blocked with 5% nonfat milk for 1 hour, washed three times with Tris-buffered saline containing 0.1% Tween 20 (150 mmol/L NaCl, 20 mmol/L Tris, pH 7.4, 0.1% Tween‐20), and then incubated overnight at 4°C with appropriate primary antibodies against phosphor-VASP^Ser157^, Akt, phosphor-Akt^Ser473^, phosphor-Akt^Thr308^, or GAPDH. Following the washing, membranes were incubated with horseradish peroxidase-conjugated anti-mouse IgG as secondary antibodies for 1 hour. The bound peroxidase activity on the PVDF membranes was detected on X-ray film using electrochemical luminescence western blotting detection system (Thermo Fisher Scientific Inc., Waltham, MA, USA) as described in the manufacturer's protocol. Optical density analysis was performed using the Image-Pro Plus software program (Media Cybernetics, USA), and the intensity of each signal was normalized to the respective intensity of *β*-actin.

### 2.10. Statistical Analysis

Data were analyzed using SPSS version 20.0 (SPSS, Inc., Chicago, USA) and the results were expressed as mean ± standard deviation (SD). For normally distributed data, statistical comparisons were performed using one-way analysis of variance followed by Dunnett's test for comparison against a single group. For non-normally distributed data, statistical analysis was performed using the Kruskal–Wallis test. Each n-value referred to the data is obtained from one donor blood sample in human platelets. GraphPad Prism 8 (GraphPad Software, San Diego, California, United States) was used for drawing figures. *P* < 0.05 was considered statistically significant.

## 3. Results

### 3.1. Effects of TMP on Platelet Aggregation

**Figure 2:**
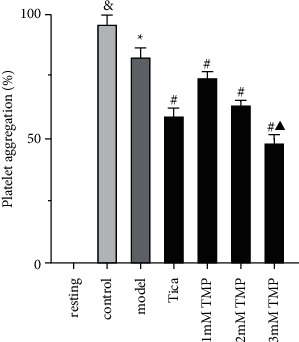
Effect of different concentrations of TMP on platelet aggregation. Results were expressed as mean ± SD. *n* = 6. ^&^*P* < 0.05 vs. resting; ^*∗*^*P* < 0.05, ^*∗*^*P* < 0.05 vs. control; ^#^*P* < 0.05, ^#^*P* < 0.05 vs. model; ^▲^*P* < 0.05 vs. Tica.

MRS2179 (100 *μ*M) showed 12.9% less platelet aggregation than the control group (*P* < 0.05; Figure 2). Using the selective P2Y_1_ antagonist MRS2179, we tested the effect of TMP on platelet aggregation mediated by P2Y_12_ receptors. In the initial screening, Tica and TMP treatment inhibited ADP-induced platelet aggregation (20 *μ*M) compared with the model group. TMP inhibited platelet aggregation in a dose-dependent manner (*P* < 0.05, Figure 2). At 3 mmol/L, TMP treatment inhibited platelet aggregation more than Tica treatment (*P* < 0.05).

### 3.2. Effects of TMP on Platelet CD62p Expression and PAC-1 Activation

**Figure 3:**
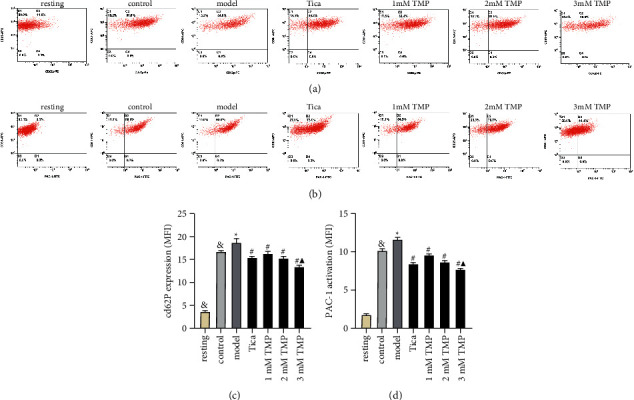
Representative images of CD62p expression (a) and PAC-1 activation (b) after interventions. Quantitative analysis of CD62p expression (c) and PAC-1 activation (d) (MFI). Results are expressed as mean ± SD. *n* = 6. ^&^*P* < 0.05 vs. resting; ^*∗*^*P* < 0.05 vs. control; ^#^*P* < 0.05 vs. model; ^▲^*P* < 0.05 vs. Tica.

After platelet activation, CD62p (P-selection), a sensitive and specific indicator for the degree of platelet activation from alpha particles, was released to the platelet membrane surface, while the PAC-1(GPIIb/IIIa receptor) shows structural changes and causes platelets to aggregate. Therefore, we examined the effect of TMP on ADP-induced platelet CD62p expression and PAC-1 activation. Pretreatment with MRS2179 increased CD62p expression and PAC-1 activation compared with the control group (*P* < 0.05, Figure 3). Tica and TMP treatment decrease CD62p expression and PAC-1 activation compared with the model group. TMP treatment gave rise to a dose-dependent decrease in CD62p expression and PAC-1 activation (*P* < 0.05). Compared to Tica, TMP at 3 mmol/L gave rise to increased inhibition of CD62p expression and PAC-1 activation (*P* < 0.05).

### 3.3. TMP Limits Clot Retraction

**Figure 4:**
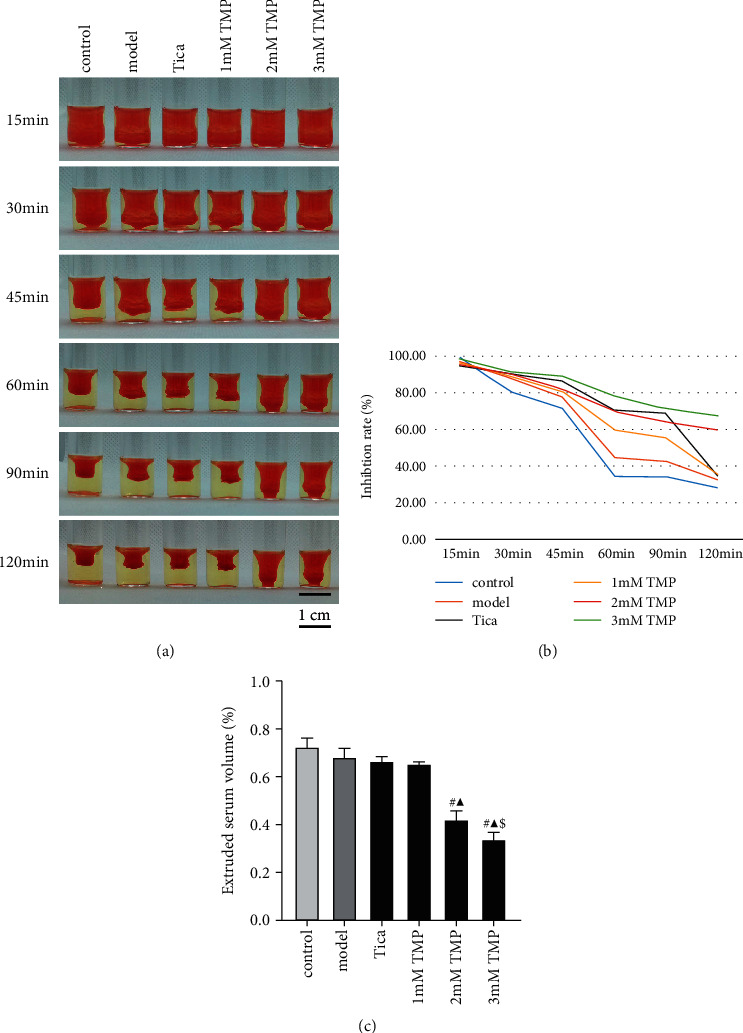
(a) Representative images of clot retraction after thrombin addition in the presence of MRS2179, ticagrelor, or various concentrations of TMP. (b)Thrombin (1 U/mL) was added to initiate fibrin clot formation, and clot retraction was observed for 120 min at room temperature. (c) The effect of TMP on clot retraction at 120 min. Results are expressed as mean ± SD. *n* = 3. ^#^*P* < 0.05 vs. MRS; ^▲^*P* < 0.05 vs. Tica; ^$^*P* < 0.05 vs. 2 mM TMP.

After activation, activated GP IIb/IIIa receptors bind to fibrinogen and cause platelet aggregation. The outside-in signaling mediated by GP IIb/IIIa receptors plays an essential role in thrombin-induced platelet activation and clot retraction. Here, we investigated the effect of TMP on clot retraction. As shown in Figure 4(a), thrombin visibly retracted the fibrin clot. The kinetics curves of the retracting clot at the indicated time points showed that intensity changes of retraction exhibited a linear increase with time (Figure 4(b)). Pretreatment with MRS2179 did not affect clot retraction in the model group compared to the control group after 120 min observation (*P* > 0.05). There was no significant difference between Tica treatment and the model group (*P* > 0.05); however, TMP treatment suppressed thrombin-induced clot retraction in a dose-dependent manner compared to the model group (Figure 4(c)).

### 3.4. Effects of SQ22536 and LY294002 on the Antiplatelet Activity of TMP Mediated by the P2Y_12_ Receptor

**Figure 5:**
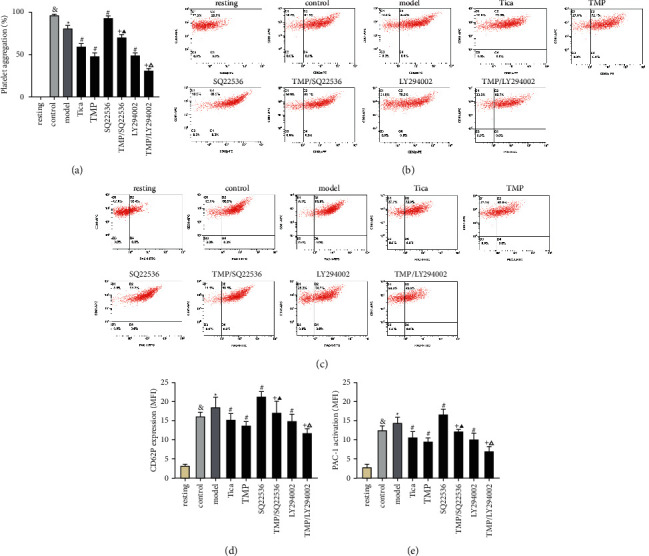
(a) Effect of SQ22536 and LY294002 on platelet aggregation. Representative images of CD62p expression (b) and PAC-1 activation (c) after different interventions. Quantitative analysis of CD62p expression (d) and PAC-1 activation (e) (MFI). Results are expressed as mean ± SD. *n* = 6. ^&^*P* < 0.05 vs. resting; ^*∗*^*P* < 0.05 vs. control; ^#^*P* < 0.05 vs. model; ^+^*P* < 0.05 vs. TMP; ^▲^*P* < 0.05 vs. SQ22536; ^△^*P* < 0.05 vs. LY294002.

ADP-related platelet activation and aggregation depend on P2Y_12_ receptor signaling, including the upregulation of PI3K/Akt phosphorylation, inhibition of AC, and subsequent decrease of cAMP. SQ22536, an adenylate cyclase inhibitor, reversed the inhibitory effect of TMP treatment on ADP-induced platelet aggregation, P-selectin expression, and GPIIb/IIIa activation compared to TMP treatment (*P* < 0.05; Figure 5). At 100 *μ*mol/L, SQ22536 reduced the inhibitory effect of TMP on ADP-induced platelet aggregation by 22.1% (*P* < 0.05, Figure 5(a)). We tested whether LY294002, a PI3K inhibitor, would reverse the inhibitory effect of TMP on ADP-induced platelet aggregation, P-selectin expression, and GPIIb/IIIa activation. We found that LY294002 enhanced these effects compared to the TMP treatment group (*P* < 0.05). At 10 *μ*mol/L, LY294002 increased the inhibitory effect of TMP on ADP-induced platelet aggregation by 16.8% (*P* < 0.05, Figure 5(a)). As a control, SQ22536 (100 *μ*mol/L) alone promoted ADP-induced platelet activation, while LY294002 (10 *μ*mol/L) alone suppressed ADP-induced platelet activation compared to the model group (*P* < 0.05).

### 3.5. Effects of TMP on Platelet Inflammatory Mediators and the Intervention Effect of SQ22536 and LY294002

**Figure 6:**
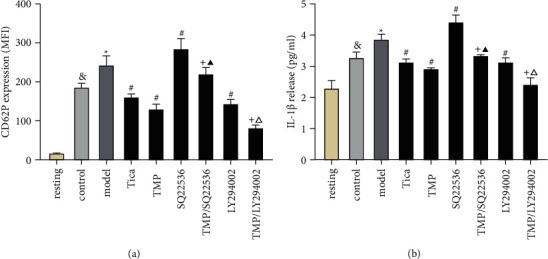
Effect of SQ22536 and LY294002 on the release of sCD40L and IL-1*β*. Results are expressed as mean ± SD. *n* = 6.^&^*P* < 0.05 vs. resting; ^*∗*^*P* < 0.05 vs. control; #*P* < 0.05 vs. model; +*P* < 0.05 vs. TMP; ^▲^*P* < 0.05 vs. SQ22536; ^△^*P* < 0.05 vs. LY294002.

The expression of platelet inflammatory mediators, sCD40L and IL-1*β*, were increased after ADP-induced platelet activationthe release of platelet inflammatory mediators (sCD40L and IL-1*β*) expression compared to the resting group (*P* < 0.05, Figure 6). Compared to the control group, the expression of sCD40L and IL-1*β* was increased in the model group, indicating that the P2Y12 receptor was overactivated by ADP (*P* < 0.05). Tica and TMP treatment showed similar inhibition of supernatant sCD40L and IL-1*β* concentrations compared to the model group (*P* < 0.05). Compared to TMP treatment, SQ22536 (100 *μ*mol/L) reversed the TMP inhibitory effect on platelet sCD40L and IL-1*β* release, while LY294002 (10 *μ*mol/L) promoted these effects. As a control, SQ22536 (100 *μ*mol/L) alone promoted ADP-induced sCD40L and IL-1*β* release, while LY294002 (10 *μ*mol/L) alone suppressed sCD40L and IL-1*β* release when compared to the model group (*P* < 0.05).

### 3.6. Effects of LY294002 on the Antiplatelet Action of TMP Mediated by Akt Phosphorylation

**Figure 7:**
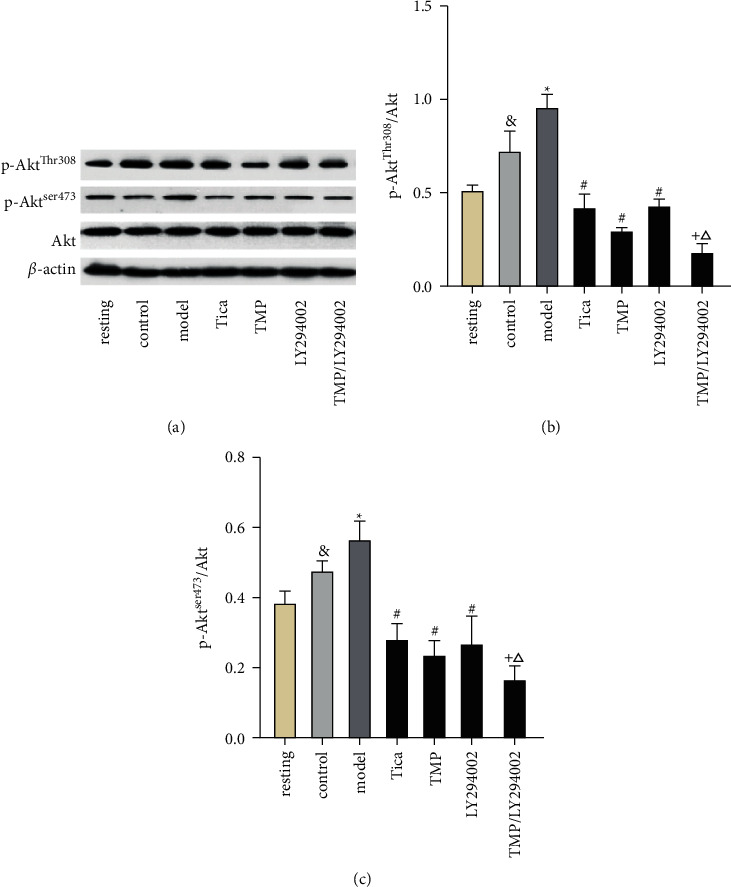
(a) Effect of LY294002 on p-Akt^ser473^ and p-Akt^Thr308^ expression of different groups evaluated by western blotting. (b, c) Results are expressed as mean ± SD. *n* = 6. ^&^*P* < 0.05 vs. resting; ^*∗*^*P* < 0.05 vs. control; ^#^*P* < 0.05 vs. model; ^+^*P* < 0.05 vs. TMP; ^△^*P* < 0.05 vs. LY294002.

Because 3 mmol/L TMP significantly inhibited ADP-induced platelet activation and aggregation, we used this concentration to investigate its effect on P2Y_12_ receptor-related signaling molecules. We found that MRS2179 activated the phosphorylation of Akt^Ser473^ and Akt^Thr308^ and a downstream molecule of P2Y12 receptor that stimulates GPIIb/IIIa activation and subsequent fibrinogen binding (Figures 7(b) and 7(c)). TMP treatment inhibited ADP-induced Akt^Ser473^ and Akt^Thr308^ phosphorylation compared to the model group. Next, we investigated whether the inhibitory effect of TMP on Akt phosphorylation depended on the PI3k/Akt pathway. As a control, LY294002 inhibited ADP-induced Akt^Ser473^ and Akt^Thr308^ phosphorylation. We found that PI3k inhibitor LY294002 promoted the inhibitory effect of TMP on Akt^Ser473^ and Akt^Thr308^ phosphorylation as compared to the TMP treatment group (*P* < 0.05).

### 3.7. Effects of SQ22536 on the Antiplatelet Action of TMP Mediated by VASP^Ser157^ Phosphorylation and cAMP Concentration

**Figure 8:**
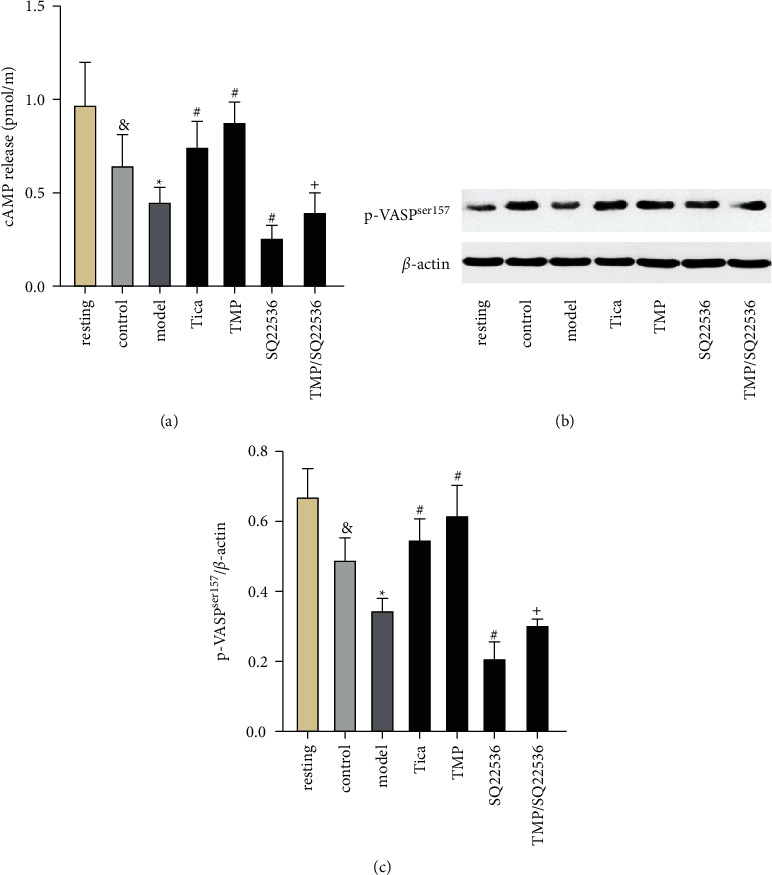
(a, c) Effect of SQ22536 on cAMP release and p-VASP^ser157^ expression. Results are expressed as mean ± SD. *n* = 6. and *P* < 0.05 vs. resting; ^*∗*^*P* < 0.05 vs. control; ^#^*P* < 0.05 vs. model; ^+^*P* < 0.05 vs. TMP. (b) Effect of SQ22536 on p-VASP^ser157^ expression various groups evaluated using western blotting.

As opposed to the phosphorylated Akt, P2Y_12_ receptor activation inhibited AC resulting in decreased cAMP concentration and inhibited VASP^Ser157^ phosphorylation. We investigated the effect of TMP on AC downstream molecules cAMP and VASP. MRS2179 decreased cAMP concentration and inhibited VASP^Ser157^ phosphorylation (*P* < 0.05; Figure 8). However, TMP treatment increased cAMP concentration and activated the phosphorylation of VASP^Ser157^ compared to the model group (*P* < 0.05). The AC inhibitor SQ22536 (the negative control) decreased cAMP concentration and suppressed VASP^Ser157^ phosphorylation. SQ22536 potently reversed the effect of TMP on cAMP concentration and VASP^Ser157^ phosphorylation when compared to the TMP treatment group (*P* < 0.05).

## 4. Discussion

We demonstrated that TMP possesses antiplatelet activity by suppressing the P2Y_12_ receptor, inhibiting ADP-induced platelet aggregation, P-selectin secretion, and GPIIb/IIIa expression, and reducing the release of atherosclerotic-related inflammatory mediators (sCD40L and IL-1*β*). The antiplatelet effects of TMP are associated with the P2Y_12_-related signaling pathway, including the upregulation of the AC/cAMP signaling pathway and the inhibition of the PI3K/Akt signaling pathway.

The P2Y_12_ receptor plays an essential role in thrombosis and antiplatelet therapies and may serve as a critical point of prevention and treatment for thrombotic diseases. Currently, clopidogrel is the most prescribed P2Y_12_ blocker for cardiovascular diseases, especially for patients after percutaneous coronary intervention. However, the antagonists do not meet ideal therapeutic requirements, and the primary limitation is drug resistance to clopidogrel. For these reasons, there remains much room for developing P2Y_12_ receptor blockers and improving antiplatelet therapy.

In the present study, we evaluated the antiplatelet activities of TMP and reported that it exhibits potent inhibitory effects on platelet activation in vitro, serving as a P2Y12 receptor blocker. TMP inhibited ADP-induced human platelet aggregation. Platelet aggregation occurs within seconds of platelet activation, and we confirmed the mechanism underlying antiplatelet activity by the marked suppression of P-selectin secretion and GPIIb/IIIa expression and activation. After activation, P-selectin is translocated from *α*-granules to the surface of the platelet membrane and determines the size and stability of platelet aggregation [20]. Simultaneously, GP IIb/IIIa is expressed as a fibrinogen receptor on the activated platelet surface and bridges adjacent platelets by binding to fibrinogen [21]. In this context, the secretion of P-selectin and the expression of GPIIb/IIIa are markers of platelet activation. In the final stabilization phase, fibrinogen binding mediates outside-in signaling, leading to clot retraction and consolidating adherent of platelets [22]. Our findings suggest that TMP inhibits these P2Y_12_ receptor-related platelet activation activities in a dose-dependent manner.

P2Y_12_ receptor stimulated by ADP contributes to the regulation of various downstream signaling pathways, one of which is the modulation of VASP phosphorylation mediated by cAMP [23]. Elevation of cAMP levels inhibited platelet activation via increased phosphorylation of VASP^ser157^ [24]. Phosphorylation of VASPser157 was associated with suppressed platelet secretion and adhesion, thereby inhibiting platelet aggregation [25]. These findings suggest that increased cAMP and VASP phosphorylation by TMP might interfere with the cAMP signaling pathway, leading to suppressed platelet activation. The importance of cAMP-mediated pathways in regulating platelet reactivity has been well established, whereas AC inhibitor SQ22536 negatively regulates the inhibitory effects of antagonists on platelet activation by suppressing the formation of cAMP [26]. In this study, we provided evidence that SQ22536 inhibited the antiplatelet activities of TMP, confirming that upregulation of the AC/cAMP signaling pathway mediates antiplatelet effects of TMP. These findings are consistent with reports that TMP has positive effects on mouse aortic atherosclerotic plaque formation in vivo [27]. TMP also participated in inhibiting Akt phosphorylation, a downstream molecule in the PI3K signaling pathway that triggers platelet activation [28]. However, the PI3K inhibitor, LY294002, attenuated the antiplatelet activity of TMP, suggesting that there may be other mechanisms in the interactions between TMP and Akt phosphorylation. Similar antiplatelet activities of TMP (also called ligustrazine hydrochloride in this study) were observed when tested in isolated rat platelets upon ADP and insulin-like growth factor-1 induced activations [29]. It is interesting to note that, in a previous study, the PI3K/Akt pathway was reported to be a potential target of TMP in activated platelets [27].

Studies reported that the release of platelet proinflammatory mediators such as sCD40L and IL-1*β* plays a pathogenic role in atherothrombosis [30, 31]. Several lines of evidence demonstrate the potent anti-inflammatory effects of TMP [32–34]. In this regard, TMP reduces platelet-related inflammation, and the P2Y_12_ signaling pathway regulates these effects.

Taken together, our findings suggest that TMP significantly stimulates cAMP production, the phosphorylation of VASP^ser157^, and the dephosphorylation of Akt, contributing to inhibition of P2Y_12_ receptor-mediated platelet aggregation, P-selectin expression, and GPIIb/IIIa activation and the release of sCD40L and IL-1*β*. These findings suggest that TMP might serve as a P2Y_12_ receptor blocker in platelet-mediated thrombotic disease that acts via platelet aggregation and inflammation inhibition. It remains unknown whether in vitro TMP-mediated antiplatelet effects are expressed in vivo; therefore, future studies should expand the investigation of TMP properties in thrombotic and bleeding events.

## Data Availability

The data used to support the findings of this study are available from the corresponding author upon request.
